# More Symmetrical Children Have Faster and More Consistent Choice Reaction Times

**DOI:** 10.1037/a0038756

**Published:** 2015-02-09

**Authors:** David Hope, Timothy C. Bates, Dominika Dykiert, Geoff Der, Ian J. Deary

**Affiliations:** 1 Centre for Cognitive Ageing and Cognitive Epidemiology, and Department of Psychology, University of Edinburgh; 2 Centre for Cognitive Ageing and Cognitive Epidemiology, University of Edinburgh, and Medical Research Council Social and Public Health Sciences Unit, Glasgow, United Kingdom; 3 Centre for Cognitive Ageing and Cognitive Epidemiology, and Department of Psychology, University of Edinburgh

**Keywords:** symmetry, fluctuating asymmetry, RT, development, system integrity

## Abstract

Greater cognitive ability in childhood is associated with increased longevity, and speedier reaction time (RT) might account for much of this linkage. Greater bodily symmetry is linked to both higher cognitive test scores and faster RTs. It is possible, then, that differences in bodily system integrity indexed by symmetry may underlie the associations of RT and intelligence with increased longevity. However, RT and symmetry have seldom been examined in the same study, and never in children. Here, in 2 large samples aged 4 to 15 (combined *n* = 856), we found that more symmetrical children had significantly faster mean choice RT and less variability in RT. These associations of faster and less variable RT with greater symmetry early in life raise the possibility that the determinants of longevity in part originate in processes influencing bodily system integrity early in the life-course.

Bodily symmetry (van Valen, 1962) has been used to assess the reliability of phenotypic development despite stress (Waddington, 1957). Greater symmetry has, in at least some cases, been shown to be linked to better health outcomes across a range of domains (Van Dongen & Gangestad, 2011). For traits that are bilaterally symmetrical, deviations from symmetry can be assessed by aggregating differences across bilateral paired structures such as ankles, digits, or locations on the face to provide an estimate of overall asymmetry in the organism, with perfect symmetry being ideal. This trait is often referred to in the literature as Fluctuating Asymmetry (FA), referring to the origins of asymmetry in deviations (fluctuations around) mean differences of zero. In the present article, for ease of understanding, we refer to symmetry rather than FA.

Symmetry increases as children approach adulthood (Wilson & Manning, 1996), before subsequently declining in old age (Kobyliansky & Livshits, 1989). If symmetry indexes the reliability or precision of developmental processes, it can provide important insight into the timing and origins of processes influencing life-course physical and cognitive development. Reaction time (RT), too, improves across childhood, and, in adulthood and older age, RT is predictive of longevity (Deary & Der, 2005a; Shipley, Der, Taylor, & Deary, 2006). Despite both RT and symmetry being linked to early development and bodily integrity, the two have seldom been assessed jointly, and never, to our knowledge, in children. Thus, the bodily integrity hypothesis (Deary, 2012) that both cognitive and physical processes linked to life-course health are in turn associated with each other has yet to be tested for these two variables. Here we examine the association of symmetry and RT in childhood, testing the system integrity hypothesis that these variables will be significantly associated in the human organism prior to aging and illness.

Previous work linking measures of bodily symmetry to cognitive ability has focused on intelligence test scores, with a meta-analytic effect size of around .16 being reported (Banks, Batchelor, & McDaniel, 2010; Bates, 2007; Prokosch, Yeo, & Miller, 2005). In addition, numerous studies have validated an association of both more rapid simple (SRT) and choice RTs (CRT) and lower RT variability with increased intelligence (Bates & Stough, 1998; Deary, Der, & Ford, 2001; Jensen, 1992; Jensen & Munro, 1979). Effect sizes for the RT–IQ association are around double the magnitude of those found for symmetry and IQ (i.e., around −.4). Importantly, both intelligence (Deary, 2008) and RT (Deary & Der, 2005a; Shipley et al., 2006) are associated with longevity—with medium or large effect sizes—and the association of intelligence with longevity is largely accounted for by RT (Deary & Der, 2005a). The finding that RT is at least as strong a predictor of mortality as tests of reasoning supports the hypothesis that intelligence and mortality are related via life-course health choices (Lynch, Kaplan, & Salonen, 1997). Both RT and intelligence have been proposed as indicators of an underlying property that has been referred to as bodily “system integrity” (Deary, 2012; Whalley & Deary, 2001). On this view, RT is viewed as a proxy for system integrity. Consistent with this idea, and similarly to mortality risk itself, RT follows a U-shaped curve across the life span: It is slower in childhood and old age, and optimal during young adulthood (Koga & Morant, 1923; Wilkinson & Allison, 1989). RT is more variable in older adults (Hultsch, MacDonald, & Dixon, 2002). Variability in RT appears to reflect different biological processes than mean RT (MacDonald, Li, & Bäckman, 2009), is an important predictor of cognitive ability in healthy adults (Deary & Der, 2005b) and predicts future risk of cognitive decline (Bielak, Hultsch, Strauss, MacDonald, & Hunter, 2010). Research on the neurobiological mechanisms that underlie intraindividual variability in RT using fMRI analyses have indicated that RT variability is associated with level of activity in the frontal regions (Bellgrove, Hester, & Garavan, 2004), whereas damage to frontal regions increases RT variability (Stuss, Murphy, Binns, & Alexander, 2003). This reflects the growing evidence that intraindividual variability in general is predictive of overall status of the organism (MacDonald, Nyberg, & Bäckman, 2006). Variability in RT may therefore reflect important biological processes relevant to system integrity, above and beyond those captured by RT alone (MacDonald et al., 2009).

If such a latent trait of system integrity exists, then markers of system integrity should associate with health, with cognitive ability, and with each other (Gale, Batty, Cooper, & Deary, 2009). Therefore, because both RT and symmetry (Benderlioglu & Nelson, 2004; Knierim et al., 2007; Manning, Koukourakis, & Brodie, 1997; Waynforth, 1998) are putative markers of system integrity, RT should in turn be linked to symmetry. An association of symmetry with faster and less variable RT in childhood, then, would further support the idea that symmetry and RT are markers of system integrity. It would also buttress the idea that some causes of life span system integrity and longevity are present early in development, prior to both adult lifestyle choices and the onset of the chronic diseases that are responsible for most adult mortality. Finally, as Gale, Batty, Cooper, and Deary (2009) argued, the utility of system integrity in scientific understanding of life-course development depends on the availability of robust markers of this construct. As both symmetry and RT are easy to measure, an association between the two in childhood would support their utility as early developmental markers in life-course research. Also, as Deary (2012) argued, testing an association between RT and symmetry is valuable for the system integrity hypothesis because they are phenotypically so different; if they are associated, this argues for a more general system integrity than does the association between, say RT and cognitive or sensory variables.

Symmetry appears to be correlated with a number of important underlying biological processes. Hormonal factors—both cyclical and long-term—are known to correlate with symmetry, (Jasienska, Lipson, Ellison, Thune, & Ziomkiewicz, 2006; Manning & Wood, 1998), while individuals with asymmetric bodies exhibit deviations from normal brain asymmetries (Thoma, Yeo, Gangestad, Lewine, & Davis, 2002). Handedness, which is linked to genetics (Vuoksimaa, Koskenvuo, Rose, & Kaprio, 2009), physical and mental health outcomes (Bryden, Bruyn, & Fletcher, 2005; Denny, 2009) and efficiency of hemispheric interactions (Cherbuin & Brinkman, 2006), correlates with symmetry (Reilly et al., 2001). The association between symmetry and important biological variables further supports the possibility that symmetry is a marker of system integrity, and that correlations between symmetry and RT deserve exploration.

As far as we are aware, only two studies have tested the links of symmetry with RT. Thoma et al. (2006) showed that higher symmetry was associated with faster simple and choice RTs in a small sample of 21 right-handed male adult subjects. The other study examined facial symmetry and RT in 216 83-year-old subjects (Penke et al., 2009). It reported that higher facial symmetry was significantly associated with faster and less variable CRT in men, but not in women, and did not correlate with SRT or SRT variability. These two studies tentatively suggest that higher symmetry may be associated with faster CRT. Sample sizes have been too small to allow strong conclusions about what are predicted to be modest effects (Banks et al., 2010). It remains unclear whether effects are restricted to CRT, or if they are simply larger in CRT than in SRT or variance measures, and whether the effects are found in females.

In the present report we examine a large (total *n* = 856) sample of children across two studies, with roughly equal numbers of males and females. Examining children in this context is especially important, as RT improves (response times become lower) during childhood alongside the overall maturation of the organism, and symmetry increases during the same period (Hope, Bates, Dykiert, Der, & Deary, 2013; Wilson & Manning, 1996). Because symmetry and RT are both suggested as indicators of system integrity (Deary, 2012), we hypothesize that more symmetrical children will exhibit faster and less variable CRTs.

## Study 1

### Participants

Participants were visitors to the 2009 Edinburgh International Science Festival. Children were tested as part of a short public science engagement exercise, and no background measures (e.g., health factors, handedness, or cognitive ability) were recorded. Here, only data for participants who completed measures of symmetry and RT are described. Four-hundred and 97 children participated and supplied usable data for symmetry and RT assessments. They were aged between 4 and 15 years (*M* = 9.41, *SD* = 2.30); 210 were males (age *M* = 9.49, *SD* = 2.34), and 287 were female (age *M* = 9.36, *SD* = 2.27). The sex ratio remained roughly equal across all age groups. Informed consent for each child’s participation was obtained from a parent or guardian. Postcode information obtained for this sample was used to examine relative social deprivation, and the results suggested that participants were relatively socioeconomically homogeneous and on average more affluent than the general population (Dykiert, Der, Starr, & Deary, 2012).

### Apparatus and Procedure

#### Symmetry

Both hands of each participant were scanned using a digital flatbed scanner, and were rescanned where poor images occurred (typically due to motion while scanning). Lengths and widths of the digits (except the thumb) were assessed with digital-image analysis software. The landmarks used for measurement were the tip and bottom finger crease of each digit for length, and the edge of each finger at the upper finger crease for width. Reliability was assessed jointly for Studies 1 and 2 and found to be high. Initially, we measured three participants who had provided two sets of hand images, to establish rater consistency. We then calculated the intraclass correlation coefficient for the two hand images. For the three participants the intraclass correlation coefficient was .993, .989 and .991, respectively. For a 25-image subsample measured twice by the first author the intraclass correlation coefficient was .997. As in most other studies in this area (Bates, 2007; Furlow, Armijo-Prewitt, Gangestad, & Thornhill, 1997), symmetry was calculated using the formula ∑|(left - right)/(left + right)/2|, which, when multiplied by 100, gives a percentage of symmetry with higher numbers indicating greater asymmetry. It also standardizes scores for traits of different sizes. The final outcome measure, the mean symmetry score, is an average of the symmetry scores for the eight traits. Because directional asymmetry—the tendency for one side of the trait to be larger than the other side (van Valen, 1962)—can confound such scores we tested for directional asymmetry of each trait via *t* test. After controlling for multiple comparisons no traits exhibited directional asymmetry. Likewise, the average size of the traits of the left hand did not differ from the average size of the traits of the right hand, *t*(496) = 1.3, *p* = .20. Testing whether the effects were due to being asymmetrical in any direction, asymmetrical in a particular direction, or both, produced similar results to the original analyses and so we reverted to the simpler models. Zero indicates perfect symmetry, with higher scores indicating relatively less symmetrical individuals.

In order to test whether or not digit ratio (Manning & Fink, 2008) was a worthwhile target for investigation, we calculated second to fourth digit ratios for all participants. As predicted by past research (Dongen, 2009; Manning, Fink, Neave, & Szwed, 2006) symmetry and digit ratios were correlated, which may reflect the possibility that digit ratios are partly caused by symmetry. As digit ratio was beyond the core scope of the project, we replicated the symmetry-RT results while excluding the symmetry measurements of the second and fourth digits (no change in significance in any model) then conducted no further work on digit ratio.

#### Reaction time

In Study 1, simple and 4-choice RT were measured using upgraded versions of the testing devices used for the U.K. Health and Lifestyle Survey (HALS); see Cox, Huppert and Whichelow (1993) for information on the HALS study and Deary et al. (2001) for more information on a version of the RT device used here. The device had a screen to present stimuli, and five response buttons labeled 0 to 4. During SRT trials, the central button (0) was operated by a finger on the preferred hand. For SRT trials, a “0” (zero) appeared in the small liquid crystal (LCD) display and participants were instructed to press the 0 button as soon as the stimulus appeared. All participants completed eight practice trials followed by 20 experimental trials. In the 4-choice CRT task, a number between 1 and 4 would appear in the LCD and the participant was instructed to press the appropriate response button. Buttons 1 and 2 were operated with the middle and index finger of the left hand, and Buttons 3 and 4 with the same fingers of the right hand. Eight practice trials were given, followed by 40 test trials. All subjects received the same sequence of CRT stimuli. On both tasks, the intertrial interval varied from 1–3 s. The tasks were presented in a fixed order: SRT preceded CRT, and response latency was recorded automatically for each trial. All data were collected by trained testers in a laboratory section of the festival. For both SRT and CRT conditions there were enough trials to achieve acceptable standards of reliability (de Hamsher & Benton, 1977).

RT data were processed as follows. Incorrect responses were excluded, along with prepresses (RT scores of zero), and responses of < 100 ms and 150 ms for SRT and CRT, respectively. Very slow responses were also excluded (for SRT > 3,000 ms, CRT > 5,000 ms). Subjects with > 25% missing trials were removed. Each year (between 4 and 15) constituted its own age band for further, age-specific exclusion criteria. Trials with RTs greater than 5 *SD* above the mean for that age group were removed, as were participants with mean scores more than three interquartile ranges above the age-specific third quartile. As a result of these screening processes 80 participants were removed and the 497 participants described above remained. Four RT outcome measures were calculated: mean SRT and 4-choice CRT, and the standard deviation for each participant’s scores across all valid trials for CRT (CRT-*SD*) and SRT (SRT-*SD*). RT scores for the four measured variables can be found in Table 1. A summary of important characteristics broken down by age and sex can be found in Table 2.[Table-anchor tbl1][Table-anchor tbl2]

### Statistical Analyses

For each RT variable we ran a stepwise multiple linear regression model. In each model we controlled for sex and then age, before testing the effect of symmetry. The effects of sex and age have been retained in the final models whether or not they were significantly associated with the RT variable. Full details can be found in Table 3. We have not corrected for multiple comparisons.[Table-anchor tbl3]

### Results

Mean symmetry across the sample was 0.70% (*SD* = 0.43). There were no significant sex differences in symmetry (male mean symmetry = 0.71% (*SD* = .44), female mean symmetry = 0.69% (*SD* = 0.42), β = 0.02, *F*(495, 1) = 0.16, *p* = .69). Sex differences in mean RT and intraindividual variability in RT scores were examined in regression models using RT scores as the outcome with sex as a predictor. There were no significant sex differences in any of these variables (maximum β = −0.05, *F*(495, 1) = 1.253, *p* = .26 for simple RT). The sexes also did not differ significantly on age (β = 0.03, *F*(495, 1) = 0.38, *p* = .54).

We next moved to test the core hypothesis, that greater symmetry would predict lower RTs. Four initial models were tested; one for each RT variable. In all four models, older children were significantly faster, and there were no sex differences in any of the models. Symmetry was significantly associated with mean CRT (β = 0.08, *p* = .03, delta *R*^2^ = .004). The association was not significant for mean SRT (β = 0.04, *p* = .20), nor for either variability measure (SRT-*SD* β = 0.01, *p* = .08 and CRT-*SD* β = 0.03, *p* = .41, respectively). Controlling for age, age^2^, age^3^, and sex did not influence the significance or direction of the effects (β = 0.05, *p* = .03, β = 0.04, *p* = .27, β = 0.03, *p* = .63 and β = 0.01, *p* = .62 for CRT, SRT, CRT-*SD* or SRT-*SD*, respectively). Significant interactions were observed between sex and age, and between sex and symmetry, for the CRT model only. Older male children, and more symmetrical male children, were slightly faster. Controlling for mean CRT/SRT when testing the association between symmetry and CRT-*SD*/SRT-*SD* did not alter either result. Full details can be found in Table 3.

The significant association for choice RT and symmetry supported the hypothesis that increased symmetry would be associated with decreased RT. However, a follow-up study was next conducted using similar methods and participant pool to test the replicability of the association of CRT with symmetry, and this second study is reported next before a combined discussion of the findings.

## Study 2

### Participants

Study 2 was conducted to test the replicability of the findings of Study 1. It was conducted at the same location (the Edinburgh Science Festival) 1 year later, in 2010. Participants were all children visiting the festival and as previously, no background (e.g., health or cognitive measures) were collected. A total of 359 children completed the symmetry and RT tasks. Participants were aged between 4 and 15 years (*M* = 9.45, *SD* = 2.13); 174 were male (age *M* = 9.36, *SD* = 2.15), and 185 were female (age *M* = 9.53, *SD* = 2.11). The sex ratio remained roughly similar throughout, with only the youngest age group (4–6) having more males than females.

### Apparatus and Procedure

Study 2 utilized a similar procedure for the collection of symmetry data to that used in Study 1. As in Study 1, no directional asymmetry effects were observed. The average size of the traits of the left hand did not differ from the average size of the traits of the right hand, *t*(358) = 1.2, *p* = .21. Rerunning the analyses while testing for the impact of being asymmetrical in any direction, a particular direction, or both, did not meaningfully alter the results, and so we reverted to the original analyses. We again calculated second to fourth digit ratios, found them to be correlated with symmetry scores, and reproduced the analyses with the symmetry scores of the second and fourth digits removed. This did not impact the significance of any model, and digit ratio was not investigated further. The RT data were collected using a new computerized RT task: a children’s version of the Deary-Liewald RT task (2011). This task has been validated against the RT box measure used in Study 1, with the version used here having stimuli oriented to engage children’s attention, with all other aspects of the task being the same as that described in Deary, Liewald, and Nissan (2011).

**Figure 1 fig1:**
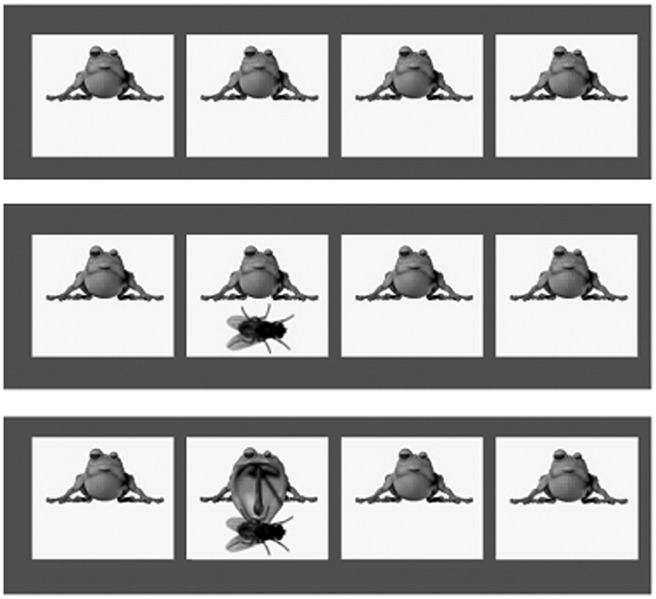
Deary-Liewald Reaction Time Task for Study 2. Note: top-most image represents prestimulus phase. No response is required. Middle image describes the stimulus phase. Here, participants would need to select the “x” key to indicate a correct response. Any other presses would be recorded as incorrect. In the bottom image, the program indicates a correct response: the frog eats the fly. This version of the Deary-Liewald task (Deary, Liewald, & Nissan, 2011) is designed for children and presents different images from those of the adult version.

The task was run on a computer monitor with a 60 Hz refresh rate. Only CRT and CRT-*SD* were recorded; SRT was not tested. Eight practice trials were given, followed by 40 experimental trials so that, as in Study 1, acceptable reliability could be achieved. On each trial, four white boxes were displayed horizontally across the middle of the screen against a dark blue background. In each box there was a frog (see Figure 1). After an interval of 1–3 s (selected randomly from this range) a fly would appear randomly in one of the four squares. This was the stimulus: The participant had to press the corresponding response key in order to complete the trial correctly. If the participant entered the correct response, the frog would appear to swallow the fly (see Figure 1, final panel). If the response was incorrect the fly would disappear. The software logging recorded the RT and whether or not the correct key was pressed for each trial.[Fig-anchor fig1]

The keys used were “z” (to select the far left square), “x” (second from left), the “comma” key (second from right) and the “full stop” key (far right). In all cases, participants rested the index and middle fingers of the left hand on the “z” and “x” keys, and the index and middle fingers of the right hand on the “comma” and “full stop” keys.

Means and *SD*s for each participant were automatically computed, with only valid trials included in the results. Prepresses (RT scores < 0 ms) were discarded, as were extremely fast (< 150 ms) and slow responses (> 5,000 ms). Participants with > 25% missing trials were removed. Trials with RTs higher than 5 *SD* above the mean for their age in years were removed, along with participants with mean scores more than three interquartile ranges above the age-specific third quartile. After these screening processes 84 participants were removed, leaving 359 participants. Means and *SD*s can be found in Table 1. The relatively faster, and somewhat more homogeneous RTs exhibited here in comparison to Study 1 also reflect the findings from adult testing comparing the present computerized task with the response box used in Study 1 (2011). A summary of important characteristics divided by age and sex can be found in Table 4.[Table-anchor tbl4]

### Sensitivity Analyses

A number of additional tests were administered to check for potentially confounding effects. To evaluate handedness, we used a “peg board” test whereby children removed and reinserted pegs from a board under timed conditions. In the first (practice) test they made use of both hands. In the second test, they used their preferred hand, and in the third test, their less preferred hand. The difference in time taken to remove and reinsert all the pegs for their preferred hand and their less preferred hand formed a measure of handedness. Zero indicated no preference, with values further away from zero indicating stronger preferences for one hand over the other. Second, we used a grip strength machine to measure the amount of pressure (in kg) the participants could apply. This was measured twice for each hand and averaged. Third, we recorded the size of the hands (as a proxy for total size). In total, 320 participants completed all the measures and 359 completed some of them. Inspection of the correlation matrix of these variables plus symmetry, age and RT indicated no associations that would indicate confounding effects. Only handedness is discussed further.

### Statistical Analyses

The statistical analyses in Study 2 mirrored those of Study 1, with a stepwise multiple linear regression model for each RT variable. We again controlled for sex and then age, before testing the effect of symmetry and retained both sex and age in the final model. Full details can be found in Table 3. We have not corrected for multiple comparisons.

### Results

Mean symmetry in the sample was 0.68% (*SD* = 0.23%). Symmetry did not differ significantly between males and females (β = −0.08, *F*(357, 1) = 2.65, *p* = .11); males = 0.70% (*SD* = 0.25), females = 0.66 (*SD* = 0.21). There were no significant differences in age between the sexes (β = 0.04, *F*(357, 1) = 0.54, *p* = .46), nor were there sex differences in CRT mean (β = 0.06, *F*(357, 1) = 0.60, *p* = .44) or intraindividual variability in CRT (β = 0.002, *F*(357, 1) = 0.002, *p* = .96). We next tested our core hypothesis that symmetry would be associated with faster, less variable RTs, especially CRTs.

For both models, older children were significantly faster. Females exhibited very slightly slower CRT than males (the only significant main effect sex difference of the six tests). Initial models using symmetry to predict CRT and CRT-*SD* indicated a significant association of (lower) mean CRT with greater symmetry (β = 0.09, *p* = .02, delta *R*^2^ = .006). Furthermore, in this sample, CRT-*SD* was also significantly associated with symmetry (β = 0.16, *p* = .001, delta *R*^2^ = .02): more symmetrical children had less variable RT. Including covariates of sex, and age and power functions of age as described above left these effects unchanged in significance and direction of effect (β = 0.07, *p* = .045; β = 0.15, *p* = .001 for CRT mean and *SD*, respectively). Controlling for mean CRT in the CRT-*SD* model did not change the significance of the results. No significant interactions between sex, age, handedness, and symmetry were observed.

## Joint Discussion of Studies 1 and 2

In both Study 1 and Study 2, we found a significant association between symmetry and RT such that more symmetrical children tended to have faster CRTs. In Study 2, but not Study 1, more symmetrical children also showed significantly less variance in CRT. In all cases, older children exhibited significantly faster RTs. In all but one model no main effect sex differences were observed. Significant interactions were observed for only one of the six models. These findings lend support to the suggestion that symmetry and RT are both markers of system integrity, and therefore the findings here are important in advancing the utility of system integrity in understanding life-course physical and cognitive change (Deary, 2012; Gale et al., 2009). Importantly, the measures of handedness used here did not influence the association between symmetry and RT in this sample, despite the potentially confounding effect of handedness with symmetry (Van Dongen, Cornille, & Lens, 2009). Although some research has suggested the existence of hormonally based sex differences in symmetry (Van Dongen & Gangestad, 2011) the lack of sex differences observed here imply this is not true, or that the differences develop later in life. The lack of interactions between symmetry and RT, and the important indicators of handedness and sex, may indicate that the association between symmetry and RT reflects basic aspects of system integrity which are independent of other aspects of bodily organization or development. It supports the proposition that the association is genuine and is not confounded by the variables of handedness or sex, though further research on behavioral handedness is required.

The significant link to choice but not simple RT is compatible with Penke et al. (2009), who reported an association of higher facial symmetry with faster and less variable CRT, but not SRT in an aged sample. Jointly, the present article and that of Penke et al. (2009) suggest that CRT may associate significantly with symmetry during childhood and old age, though further research on the adult population (i.e., age 16–82) is needed to evaluate whether the association exists across the life span.

Of three studies now available examining the link of simple RT to symmetry—Study 1, Penke et al. (2009), and Thoma et al. (2006)—only Thoma et al. (2006) found a significant association between these two variables. The two null effects (Study 1, Penke et al., 2009) both used a dedicated simple RT device. By contrast, Thoma et al. (2006) used a manual response box, which involved the respondent in raising their hand. It is possible, then, that this difference in methods may explain the difference in findings, with the Thoma procedure involving an element of complexity or cognitive control not present in the box-based, more conventional SRT procedure used in our Study 1. Furthermore, the Thoma et al. (2006) study had a small sample size, and as such the results of that study should be treated cautiously. This would suggest that responses in Thoma et al. (2006) are more comparable to the choice RT paradigm, where significant links have now been reported in four studies: the two present studies as well as Penke et al. (2009) and Thoma et al. (2006). Alternatively, the relationship between SRT and symmetry may be restricted to adolescence and early adulthood (i.e., the period of lifetime optima for both measures). Power issues may also be relevant: correlations of CRT with IQ tend to be larger than those for SRT, reflecting the increased complexity of information processing involved in each response (Jensen, 1998). It is also important to note that the direction of the effect was the same for SRT, SRT-*SD*, CRT and CRT-*SD*, and effect sizes were broadly similar (see Table 1): replication with larger sample sizes will clarify whether the association between SRT and symmetry is weaker or genuinely not present. Given that only three groups are described here, each with a very different sample mean age, and somewhat different methods, the causes of these differences cannot be identified with certainty.

Unlike Penke et al. (2009), the present results were significant in both sexes, rather than in males only (Thoma et al., 2006, did not examine females). In the study by Penke et al. (2009), the sample subjects’ mean age was 83 years and significant sex-linked attrition effects occur due to sexual dimorphism in mortality. As our sample is larger, it seems plausible that the symmetry-CRT link is present in both sexes.

Studies on mortality and cognition have tended to report medium to large associations between mortality and RTs (Deary & Der, 2005a). In the present study, the association between age and RTs were also medium to large (β = −.49 to −.77), which reflects findings in past research (Deary & Der, 2005b). By contrast, associations between symmetry and RTs were, where significant, of small effect size (β = .08 to .16). The large effect of age in the model suggests that if, there is significant measurement error, it is restricted to the measurement of symmetry alone, rather than RT. Alternatively, the relatively limited number of traits, or the limited number of measurements, may have reduced the size of the effect. It is possible that both symmetry and RT are only weak markers of system integrity, and that many measures of system integrity are needed to provide a robust measurement of that trait. Although the effect is small, this research supports a link between symmetry and RT, and consequently supports the prospect that they both measure an underlying trait of system integrity.

A strength of the present studies was their large sample sizes. In addition, homogeneity of socioeconomic status (SES) reduced the likelihood that the results reflect an unmeasured confounding variable altering both symmetry and RT. However, because symmetry is linked to SES (Hope, Bates, Penke, et al., 2013; Özener & Fink, 2010), this limited SES range may also artificially reduce the effect size, so the values found in the present studies may underestimate the population effect sizes. As few children were included in the ages entering adolescence and the reproductive period it would be of value to extend data collection into this range, especially given evidence that both symmetry (Hope, Bates, Dykiert, et al., 2013; Wilson & Manning, 1996) and RT (Hope, Bates, Penke, et al., 2013; Waynforth, 1998) reach optimal values at this time. Equally, it would be useful to explore further the symmetry and RT associations in the elderly. If symmetry and RT associate equally strongly across the life-course this would further support the proposal that they are indicating a stable life-course trait of system integrity. If, however, the magnitude of the relationship varies over the life-course, this would suggest that symmetry and RT are associated with different underlying traits.

The studies have limitations. The homogeneity of the sample with respect to socioeconomic status and nationality means it is uncertain how the effects generalize to other groups. The RT and symmetry measures were—necessarily given the testing environment—brief and covered only essential measurements, though they were sufficient to achieve acceptable levels of reliability (de Hamsher & Benton, 1977). More thorough and detailed measures, such as more RT tests, or symmetry measurements using the face or body as well as the hands, would increase reliability and would help clarify if the SRT association is nonexistent, or just weaker than the CRT associations. As behavioral handedness (i.e., handedness for tasks such as writing) was not available in these data an important opportunity remains for further experiments. In particular, relations of behavioral handedness to factors such as sex and to cerebral and functional asymmetry and underlying hormonal signals should be explored to understand how these link to hormonal explanations of symmetry (Jackson, 2008). As handedness may induce size differences in traits (e.g., the dominant hand may be a different size due to more frequent use), controlling for behavioral handedness will further increase the reliability of symmetry measurements (Van Dongen et al., 2009).

Lastly, no additional cognitive measures were recorded, which, given associations between symmetry, RT, and intelligence, would have been helpful in evaluating why the association between symmetry and RT exists. Further research incorporating other cognitive tests would be a useful advance on the present work.

In summary, these findings provide empirical support for the hypothesis that RT and symmetry both indicate underlying bodily system integrity. Symmetry may indicate system integrity by reflecting the total stress received by the organism, or the organism’s capacity to follow their original genetic “blueprint” in a precise way. Importantly, the fact that symmetry and RT are associated so early—as young as age 4—indicates that relationships seen later in life are not due entirely to illness or injury in adulthood, or by differences in lifestyle or access to health and educational facilities during late childhood or adulthood. The correlation of two markers associated with system integrity during childhood suggests that cognitive and physical abilities across the life-course are, at least in part, influenced by processes in early development.

## Figures and Tables

**Table 1 tbl1:** Means, SDs for Reaction Time (RT) Scores for Study 1 and Study 2

	Subjects	Simple RT (ms)		Simple RT—*SD* (ms)		Choice RT (ms)		Choice RT—*SD* (ms)	
		Mean	*SD*	Mean	*SD*	Mean	*SD*	Mean	*SD*
Study 1	497	366	82	102	58	776	223	184	99
Study 2	359	NA				673	179	159	56

**Table 2 tbl2:** Summary of Key Variables in Study 1 Grouped by Age

Age	Males						Females					
	Number	Symmetry	Simple RT	Simple RT-*SD*	Choice RT	Choice RT-*SD*	Number	Symmetry	Simple RT	Simple RT-*SD*	Choice RT	Choice RT-*SD*
4–6	35	.90	426	148	1,088	313	47	.70	456	169	1,121	328
7–9	89	.70	380	107	803	186	134	.68	378	98	790	182
10–12	67	.63	317	84	609	123	88	.74	328	80	633	127
13–15	19	.64	304	63	565	122	18	.60	278	56	555	104
All	210	.71	361	103	768	184	287	.69	369	101	782	184
*Note.* Symmetry is measured as a percentage. Zero indicates perfect symmetry. All RT variables are measured in milliseconds. RT = reaction time.												

**Table 3 tbl3:** Outputs of Final Regression Models for Study 1 and 2

	Study 1			Choice RT-*SD*	Study 2	
	Simple RT	Simple RT-*SD*	Choice RT		Choice RT	Choice RT-*SD*
Sex	−.001 (.01) −.03	.003 (.01) .03	−.004 (.001) −.01	.004 (.02) .02	.03 (.01) .08*	.004 (.005) .04
Age	−.02 (.001) −.58***	−.01 (.001) −.49***	−.07 (.002) −.79***	−.03 (.001) −.66***	−.06 (.00) −.72***	−.01 (.001) .56***
Symmetry	.001 (.001) .04	.001 (.001) .01	.05 (.01) .08*	.01 (.01) .03	.07 (.03) .09*	.04 (.01) .16***
Interaction between sex and age			.02 (.01)*			
Interaction between sex and symmetry			.03 (.01)*			
Adjusted *R*^2^	.33	.25	.61	.45	.54	.35
Delta *R*^2^	.001	.004	.004	.001	.006	.02
*Note.* Values are B(SE) with standardized β below. * = significance at the .05 level; ** = significance at the point .01 level; *** = significance at the .001 level; RT = reaction time. Delta *R*^*2*^ values refer to the effect of dropping symmetry. As standardized β values are less interpretable for interactions, they are not reported.						

**Table 4 tbl4:** Summary of Key Variables in Study 2 Grouped by Age

Age	Males				Females			
	Number	Symmetry	Choice RT	Choice RT-*SD*	Number	Symmetry	Choice RT	Choice RT-*SD*
4–6	26	.84	879	201	17	.70	962	216
7–9	81	.71	697	170	93	.63	738	175
10–12	58	.65	560	133	65	.68	560	131
13–15	9	.49	458	93	10	.68	465	98
All	174	.70	667	159	185	.66	681	159
*Note.* Symmetry is measured as a percentage. Zero indicates perfect symmetry. All RT variables are measured in milliseconds. RT = reaction time.
